# Computerized Symbol Digit Modalities Test in a Swiss Pediatric Cohort Part 1: Validation

**DOI:** 10.3389/fpsyg.2021.631536

**Published:** 2021-04-22

**Authors:** Céline Hochstrasser, Sarah Rieder, Ursina Jufer-Riedi, Marie-Noëlle Klein, Anthony Feinstein, Brenda L. Banwell, Michelle Steiner, Li Mei Cao, Karen Lidzba, Sandra Bigi

**Affiliations:** ^1^Division of Child Neurology, Department of Pediatrics, University Children’s Hospital Bern, University of Bern, Bern, Switzerland; ^2^Department of Psychiatry, University of Toronto, Toronto, ON, Canada; ^3^The Children’s Hospital of Philadelphia, Perelman School of Medicine, University of Pennsylvania, Philadelphia, PA, United States; ^4^Institute of Social and Preventive Medicine, University of Bern, Bern, Switzerland; ^5^Department of Neurology, University Hospital Bern, University of Bern, Bern, Switzerland

**Keywords:** SDMT, c-SDMT, information processing speed, sex-effect, age-effect, norms, test-retest reliability, practice effects

## Abstract

**Objective:**

The objective of this study was to validate the computerized Symbol Digit Modalities Test (c-SDMT) in a Swiss pediatric cohort, in comparing the Swiss sample to the Canadian norms. Secondly, we evaluated sex effects, age-effects, and test–retest reliability of the c-SDMT in comparison to values obtained for the paper and pencil version of the Symbol Digit Modalities Test (SDMT).

**Methods:**

This longitudinal observational study was conducted in a single-center setting at the University Children’s Hospital of Bern. Our cohort consisted of 86 children (45 male and 41 female) aged from 8 to 16 years. The cohort included both healthy participants (*n* = 38) and patients (*n* = 48) hospitalized for a non-neurological disease. Forty eight participants were assessed during two testing sessions with the SDMT and the c-SDMT.

**Results:**

Test–retest reliability was high in both tests (SDMT: ICC = 0.89, c-SDMT: ICC = 0.90). A reliable change index was calculated for the SDMT (RCIp = −3.18, 14.01) and the c-SDMT (RCIp = −5.45, 1.46) corrected for practice effects. While a significant age effect on information processing speed was observed, no such effect was found for sex. When data on the c-SDMT performance of the Swiss cohort was compared with that from a Canadian cohort, no significant difference was found for the mean time per trial in any age group. Norm values for age groups between 8 and 16 years in the Swiss cohort were established.

**Conclusion:**

Norms for the c-SDMT between the Swiss and the Canadian cohort were comparable. The c-SDMT is a valid alternative to the SDMT. It is a feasible and easy to administer bedside tool due to high reliability and the lack of motor demands.

## Introduction

Information processing speed (IPS) reflects the efficiency of cognitive function (Low et al., 2017). As a fundamental cognitive function it is linked to everyday activities (Hale, 1990; Roivainen, 2011) and can be specified as a combination of the encoding of information, its transformation and retrieval, as well as perception, working speed, and attention (Weiss et al., 2015; Scharfen et al., 2018). IPS is affected by numerous factors, such as age (Gilmore et al., 1983; Yeudall et al., 1986; Emmerson et al., 1989; Bowler et al., 1992; Feinstein et al., 1994; Uchiyama et al., 1994; Richardson and Marottoli, 1996; Fry and Hale, 2000), sex (Smith, 1973; Laux and Lane, 1985; Polubinski and Melamed, 1986; Yeudall et al., 1986; Jinabhai et al., 2004; Jorm et al., 2004), and general intelligence (Yeudall et al., 1986; Nielsen et al., 1989; Uchiyama et al., 1994; Roivainen, 2011). Furthermore, it is known to vary from one person to another, as a consequence of variations in neural speed, neural efficiency, and capacity (Birren and Fisher, 1995; Mendelson and Ricketts, 2001).

Information processing speed can be used as a measure for cognitive dysfunction. In a clinical environment, impaired IPS is related to cognitive change in old age, developmental disorders, psychiatric disorders, pathological conditions of the nervous system, and to neurological injuries (DeLuca and Kalmar, 2013), such as multiple sclerosis (Bigi et al., 2017), brain tumors (Gehrke et al., 2013), or epilepsy (Reilly et al., 2015) to name but a few. IPS can be used for predicting progression or recovery in conditions like traumatic brain injury, where processing speed plays a significant role in the mediation of the correlation between the severity of the injury and post-traumatic brain injury adaptive functioning (Rassovsky et al., 2006).

Monitoring IPS is therefore an essential aspect of neurorehabilitation. A standardized test to measure IPS in pediatric populations is much needed. The ability to repeatedly assess IPS during recovery could allow rehabilitation strategies to be tailored specifically to individuals and thereby assist in the gradual return to everyday life.

Information processing speed can be assessed during different tasks varying from simple choice reaction to more complex tests, such as mental rotation (Hale, 1990). The test results express IPS in terms of time or number of correct responses in a given period of time (Sweet, 2011). Most assessments require a motor (e.g., written) or oral (e.g., spoken) response (Weiss et al., 2015). It is desirable to define testing methods that isolate impairment of IPS from other cognitive impairments (Myerson et al., 1990; Salthouse, 1996). This is why most popular IPS measurements such as naming tasks, letter comparison, box completion, digit copying, digit-symbol substitution or coding (Wechsler, 1991; Earles and Salthouse, 1995; Kemp, 2011) do not assess higher-level cognition (Fry and Hale, 2000).

Performance on IPS tests generally shows the speed and accuracy with which a participant performs a specific task (e.g., naming, coding, visual identification, simple math). Thereby it reflects the efficiency of process automaticity, information accessibility, information intake and processing (visual or auditory) (Weiss et al., 2015). However, these tests still require other cognitive functions, such as goal maintenance, filtering background information (Lustig et al., 2006), working memory, (Salthouse and Babcock, 1991; Luciana and Nelson, 1998; Eastwood, 2001) and especially decision-making (Bunce and Macready, 2005; Weiss et al., 2015). Therefore, they test not only IPS, but also different aspects of executive control (Cepeda et al., 2013) that are measured to varying extents depending on the task.

Several tests to assess IPS in a clinical setting are available for adults and for children. Children’s tests include the two subtests of the Processing Speed Index of the Wechsler Intelligence Scale for Children (WISC) (Peterman and Peterman, 2014) and the Symbol Digit Modalities Test (SDMT) (Smith, 1973).

The SDMT is one of the most popular tests for evaluating IPS in the clinical setting (Silva et al., 2018); for example, it is part of The Brief Repeatable Battery of Neuropsychological Tests and the Minimal Assessment of Cognitive Function in Multiple Sclerosis (Fittipaldi-Márquez et al., 2017).

Although it appears an easy task, execution of the SDMT demands the involvement of an astonishing complexity of cerebral mechanisms and different areas. Various key neurocognitive functions, such as attention, visual scanning, and motor speed, are required to solve the test (Sheridan et al., 2006). The strong involvement of cuneus, precuneus and cerebellum, as well as regions of the frontoparietal attentional network and occipital cortex in the performance of the SDMT were shown in a recent meta-analysis with magnetic resonance imaging (Silva et al., 2018).

Since the SDMT requires information exchange between distant brain regions rather than the involvement of isolated brain regions, it is suitable for assessing IPS (Fittipaldi-Márquez et al., 2017). The test’s layout does not permit the use of different strategies for its execution, and therefore allows a more isolated assessment of IPS than in tests with other designs (Silva et al., 2018). Advantages of the SDMT include its short duration, its inexpensiveness, ease of administration and sensitivity to numerous neuropsychiatric conditions, although it cannot specify which disorder is affecting the subject (Smith, 1973; Nocentini et al., 2006; Koh et al., 2011; Tang et al., 2018). It is reasonably reliable, with an intraclass correlation coefficient (ICC) varying between 0.72 and 0.98 (Koh et al., 2011; Pereira et al., 2015; Tang et al., 2018). Due to its sensitivity to change in neurocognitive function, the SDMT is well-suited to track disease progression (Kiely et al., 2014).

However, the normative values of the SDMT are affected by several factors such as age, sex, education, cultural background and health and therefore cannot be applied to every population (Kiely et al., 2014). Furthermore, it is impossible to combine the test with neuro-imaging, since participants cannot perform the test while in a magnetic resonance scanner (Akbar et al., 2011). Like most tests for IPS, the written format of the SDMT demands visuospatial processing and relies heavily on motor function (Low et al., 2017). This is problematic, since motor function is often impaired in patients with neurologic conditions. Parallel versions of the SDMT are provided in the brief repeatable neuropsychological battery, thus preventing practice effects (Rao, 1991; Hinton-Bayre and Geffen, 2005; Koh et al., 2011; Benedict et al., 2012; Pereira et al., 2015; Scharfen et al., 2018). Furthermore, statements about behavior of IPS during the test, like fluctuations of speed or attention, are not possible.

To address some of the limitations, alternative versions of the SDMT have been developed. For example, the computerized Symbol Digit Modalities Test (c-SDMT) used in the present study requires verbal responses rather than motor ones, and therefore reduces the motor component (Bigi et al., 2017).

In the c-SDMT subjects have to make nine verbal symbol digit pairings per trial according to a key on-screen. The test is composed of eight consecutive trials and the time needed to complete each trial is measured. This structure allows assertions about the behavior of IPS during the test. Bigi et al. (2017) assessed the c-SDMT for the first time in the pediatric population and found, that while the performance of healthy children improves significantly from trial one to trial eight, this effect is considerably smaller in children with multiple sclerosis and therefore these effects might differ depending on the neurological condition. Furthermore, in the c-SDMT the symbol–digit key can be changed from test to retest, which should minimize practice effects (Bigi et al., 2017). For children with motor impairment, this provides a new option to repeatedly test IPS.

The c-SDMT has already been validated for clinical use with adult multiple sclerosis patients. The validation study found high retest reliability for the c-SDMT in adult cohorts (ICC = 0.97) and higher sensitivity, but slightly less specificity than in the written SDMT. It has therefore been suggested, that the SDMT should be replaced by the c-SDMT for assessing patients with multiple sclerosis (Akbar et al., 2011).

A study in Canada also found high retest reliability (ICC = 0.91) for the c-SDMT in a cohort of healthy adolescents as well as in pediatric multiple sclerosis patients (Bigi et al., 2017). But before using the c-SDMT globally, further validation studies are needed, since normative differences might occur when making cross-cultural comparisons due to cultural and linguistic factors (van de Vijver and Tanzer, 2004; Cores et al., 2015). Such cultural effects have previously been shown in more complex tests like the Conners 3^®^ Rating Scales for assessing Attention Deficit Hyperactivity Disorder (Christiansen et al., 2016) but they have also been suggested to influence assessments of more fundamental functions, including IPS (Jensen, 1988).

The aim of this study was to validate the c-SDMT in a Swiss pediatric cohort by comparing the results of the Swiss sample to the Canadian norms as presented in Bigi et al. (2017). The second aim was to investigate the differential effects of sex and age on IPS as measured by the c-SDMT and the already established SDMT. We expected not only to observe increasing IPS with age, but also a marked increase in younger children during performance of the c-SDMT (i.e., children are significantly faster at the end of the eight trials than at the beginning) compared to the older child (H1). Secondly, we expected a significant sex-effect on IPS, with females performing better than males, which would be observable in their results in both the SDMT and c-SDMT (H2). Thirdly, we expected the c-SDMT to have a higher test-retest reliability than the SDMT (H3a). We expected younger children to show less test-retest reliability than older children (H3b).

## Materials and Methods

### Study Design

The study conducted was a single-center pilot study with both cross-sectional and short-term longitudinal components. It took place at the University Children’s Hospital of the University of Bern in Switzerland. The study was approved by the ethics board of the canton of Bern (project ID 2018-00540) and conducted in accordance with the ethical principles of the Declaration of Helsinki. Recruitment and testing of participants started in July 2018 and ended in December 2018. Data collection and analysis took place between January and June 2019.

### Participants

A total of 86 children (45 male and 41 female) aged from 8 to 16 years were recruited. To minimize possible confounding by an influence of a hospital environment, both healthy participants (*n* = 38) and children and adolescents hospitalized for a non-neurological disease (*n* = 48) were tested. A subsample of 48 children returned for retest.

Outpatient participants were recruited through flyers distributed at the hospital clinics, in private pediatric practices, sports clubs and schools, as well as through the hospital website. Inpatient participants were identified by different departments of the University Children’s Hospital Bern and invited to participate by a member of the study team.

Interested individuals were screened for eligibility. Those included were children aged 8 to 16 years who were either healthy or had been hospitalized with a non-neurological disease. All participants were native German speakers. Exclusion criteria were: medication with known psychotropic effects, diagnosis of attention deficit disorder, anxiety, autism or depression, visual impairment such that the test could not be completed, history of traumatic brain injury, and lack of consent. For comparative reasons we referred to the data on the Canadian standardization of the c-SDMT, as published in Bigi et al. (2017).

### Procedures

#### SDMT

The written form of the SDMT consists of a sheet of paper with a key of nine symbols. Each symbol is matched to a corresponding number from 1–9. Below the key, participants are presented with a total of 120 randomly ordered symbols to which they are asked to match the digits in writing. The task is to match as many digits to the symbols as possible in 90 s. The score is the number of correctly matched symbol–digit combinations accomplished in that time. The more correct pairings the subjects makes within 90 s, the better the score. The test was conducted according to the SDMT User Manual (Smith, 1973).

#### c-SDMT

**FIGURE 1:**
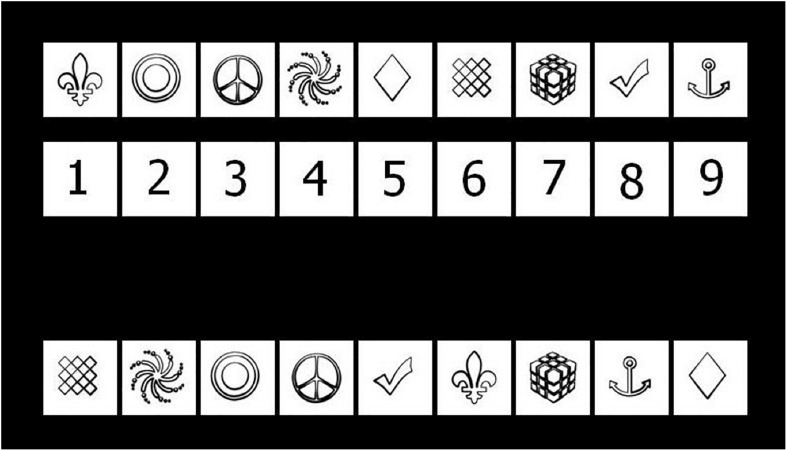
Screen capture of the c-SDMT with the key shown above and the first line of testing below. Participants are tasked with matching each symbol to the corresponding digit.

The c-SDMT is administered on a computer. Participants are presented with a key to nine symbol–digit pairings, similar to the key to the SDMT, on the upper half of the computer screen (Figure 1). A mouse click by the examiner presents the participant with randomly arranged symbols to which they are asked to verbally match the digits, while the key is still being shown on the screen. Each trial is ended with a mouse click by the examiner, as soon as the participant has finished matching the numbers to the symbols. The next trial begins automatically. The test consists of eight consecutive trials, displaying nine symbols each. The total time taken to complete all eight trials, the time needed for each single trial, and the average time needed to complete a single trial (= mean time per trial) are recorded. The examiner documents any incorrect matches. While there is no time limit on this test, approximately 5 min are needed on average to complete the full c-SDMT.

### Testing Procedure

All participants were assessed with the same tasks in the same order, starting with the TONI-4 [Test of Nonverbal Intelligence, Fourth Edition (Brown et al., 2010)], which was used to assess the IQ, then the SDMT, and lastly the c-SDMT. Inpatient children were tested in their hospital room and healthy participants were tested in a testing room at the University Children’s Hospital of Bern. The first appointment lasted for about 30 min. Retesting took place after 2 weeks ± 2 days with the SDMT followed by the c-SDMT, taking about 15 min altogether. All examiners were trained to administer the tests in the same way and standardized instructions for all tests were given.

### Statistics

Statistical analyses were conducted with IBM SPSS Statistics Version 26.0. A *p*-value ≤0.05 was considered statistically significant. For the SDMT, the final score (number of correct symbol–digit pairings in 90 s) and for the c-SDMT the mean time per trial in seconds was used.

For calculations of reliability and retest effects, the 48 participants with two testing session were included. For analyses of reliability, test–retest effects and within-test dynamics, the data was split into two age groups (younger group aged 8 to 11 years and older group aged 12 to 16 years).

To estimate reliability of the two measurement (c-SDMT and SDMT), the ICC through analysis of variance (ANOVA), was calculated to show correlation and agreement between test and retest. An ICC below 0.40 was considered poor, between 0.40 and 0.59 fair, between 0.60 and 0.74 good, and above 0.75 was considered excellent (Cicchetti, 1994). To assess test–retest effects, a one-tailed paired sample *t*-test was performed and the size of the practice effect was evaluated using Cohen’s criteria (Cohen, 1988): 0.21–0.49 indicated a small; 0.50–0.79, medium; and more than 0.80, a large effect size. The 90% confidence interval of the reliable change index for practice effects (90% CI RCIp) was calculated based on measurement error to describe the range in which changes can be considered clinically relevant.

$$90\%CIRCIp=\mathrm{mean}\mathrm{practice}\mathrm{effect}\pm 1.645\times SE_{diff}$$

$$SE_{diff}={\sqrt{2\left(SE_{m}\right)}}_{2}$$

$$SE_{m}=SD_{1}\left(\sqrt{1-ICC}\right)$$

where SE_m_ is the standard error of measurement, SE_diff_ the standard error of the differences and *SD*_1_ the standard deviation of the first measurement (Koh et al., 2011). Furthermore, a one-tailed paired *t*-test was performed to determine the mean difference between the two measurements. Convergent Validity between SDMT total score and cSDMT mean time per trial was assessed by Pearson’s correlation. Age (years) was correlated with cSDMT (mean time per trial) and SDMT (total score) performance by Pearson’s correlations.

To assess the effect of age (younger group versus older group) and sex (male versus female) on IPS, an analysis of covariance (ANCOVA) was applied to both the SDMT and the c-SDMT results. A repeated measures ANOVA with age as a between-subject factor was performed to determine whether there was a statistically significant change in the time needed per trial from the first to the eighth trial (i.e., within-test dynamics) and whether such effects differed between the two age groups. Finally, normative values were calculated for all trials in both the Swiss and the Canadian cohort (Bigi et al., 2017) and an independent-samples *t*-test for the two samples was performed to compare the cohorts and to further validate our data.

## Results

### Demographic Data

The total sample (*n* = 86) comprised 41 females and 45 males. A 48 of them returned for retest (Table 1). The results of all completed tests were evaluable and valid. There were no drop-outs.

**TABLE 1:** Demographic data of participants who were retested and those who were lost to follow-up.

	Retest *n* = 48 (56%)	Lost to follow-up *n* = 38 (44%)	*t*-test or *x*^2^	*p*-value
Age in years, mean (*SE*)	11.60 (0.35)	12.13 (0.40)	*t* = 1.00	0.32
**Sex, *n* (%)**			*x*^2^ = 1.57	0.21
Female	20 (41.7%)	21 (55.3%)		
Male	28 (58.3%)	17 (44.7%)		
**Group, *n* (%)**			*x*^2^ = 22.26	<0.001
Outpatient	32 (66.7%)	6 (15.8%)		
Inpatient	16 (33.3%)	32 (84.2%)		
**Handedness, *n* (%)**			*x*^2^ = 0.81	0.67
Right	43 (89.6%)	35 (92.1%)		
Left	4 (8.3%)	3 (7.9%)		
Both	1 (2.1%)	0 (0%)		
Non verbal IQ, mean (*SE*)	108.04 (1.61)	101.97 (1.47)	*t* = −2.79	0.01

*n = subsample size; SE = standard error; t = t-test; x^2^ = chi-squared test.*

The participants who performed the retest and those who were lost to follow-up did not differ in terms of age (*t* = 1.00, *p* = 0.32), sex (*x*^2^ = 1.57, *p* = 0.21) or handedness (*x*^2^ = 0.81, *p* = 0.67). IQ, however, was higher in the retest group (*t* = −2.79, *p* = 0.01). A significantly higher proportion of inpatients than outpatients were lost to follow-up (*x*^2^ = 22.26, *p* < 0.001). However, there was no significant difference between the outpatient and the inpatient group, regarding age, sex and nonverbal IQ (Table 1, Manuscript Part 2). The Canadian sample consisted of 450 participants between 8 and 16 years (Table 5; Bigi et al., 2017).

### Reliability and Test–Retest Effects

Both the SDMT and the c-SDMT showed excellent test–retest reliability over all participants, with an ICC of 0.89 (95% CI = 0.46, 0.96) for the SDMT and an ICC of 0.90 (95% CI = 0.35, 0.97) for the c-SDMT (Table 2). When comparing age groups, the lowest ICC = 0.64 was achieved in the c-SDMT of the older group and the highest ICC = 0.85 in the c-SDMT of the younger group. These ICC values still indicate good test–retest reliability.

**TABLE 2:** Parameters of test–retest reliability for the SDMT and the c-SDMT.

	Mean PE	*SD*_1_	ICC	*SE*_m_	*SE*_diff_	95% CI RCIp	
						Lower	Upper
SDMT, all with follow-up	5.42	11.38	0.89	3.69	5.22	−3.18	14.01
SDMT older group	5.75	7.18	0.75	3.62	5.12	−2.68	14.18
SDMT younger group	5.08	7.04	0.77	3.34	4.73	−2.69	12.86
c-SDMT, all with follow-up	−1.99	4.63	0.90	1.49	2.10	−5.45	1.46
c-SDMT older group	−1.59	2.14	0.64	1.28	1.82	−4.58	1.40
c-SDMT younger group	−2.39	4.44	0.85	1.74	2.45	−2.69	1.65

*Mean PE, mean practice effect; SD_1_, standard deviation of the first test; ICC, intraclass correlation coefficient; SE_m_, standard error of measurement; SE_diff_, standard error of difference; 95% CI RCIp = 95% confidence interval of the reliable change index for practice effects; SDMT, Symbol Digit Modalities Test; c-SDMT, computerized Symbol Digit Modalities Test. SDMT values are given as scores and c-SDMT values are in seconds.*

The paired samples *t*-test for the SDMT showed a statistically significant practice effect (PE) at retest (*p* < 0.001) with a mean PE of 5.42 Points (95% CI = 3.75, 7.08 Points) across all participants. Similar results were obtained with the younger (mean PE = 5.08 Points, 95% CI = 2.74, 7.43 Points) and older group (mean PE = 5.75 Points, 95% CI = 3.22, 8.28 Points).

A statistically significant PE (*p* < 0.001) was also demonstrated for the c-SDMT over all participants (mean PE = −1.99 s, 95% CI = −2.54, −1.44 s) as well as in both age groups (younger group: mean PE = −2.39 s, 95% CI = −3,30, −1.48 s; older group: mean PE = −1.59 s, 95% CI = −2.23, −0.96 s). Cohen’s effect size criteria showed that these PEs were small for both the SDMT (*d* = 0.45) and the c-SDMT (*d* = 0.47). Nevertheless, these PEs are clinically significant since they are above the calculated RCIp for both the SDMT (RCIp = −3.18, 14.01) and the c-SDMT (RCIp = −5.45, 1.46) (Table 2).

Convergent validity between SDMT and c-SDMT was highly significant (*r* = −0.794, *p* < 0.0001).

### Effects of Age and Sex in the SDMT and c-SDMT

Age correlated highly significantly with cSDMT and SDMT performance (cSDMT mean time per trial: *r* = −0.702; SDMT total score: *r* = 0.715; both *p* < 0.0001). The ANCOVA (Table 3) showed a significant difference between the results of the SDMT for the younger and older age group, *F*(1,83) = 77.33, *p* < 0.001, partial η^2^ = 0.48, whereas for the covariate “sex,” no significant effect could be shown, *F*(1,83) = 2.65, *p* = 0.11, partial η^2^ = 0.03. The findings of the ANCOVA for the c-SDMT were similar, showing that the average time needed per trial differed significantly between the younger and older age group, *F*(1,83) = 59.70, *p* < 0.001, partial η^2^ = 0.42. Again, the covariate “sex” did not significantly influence the average time needed per trial in the c-SDMT, *F*(1,83) = 2.99, *p* = 0.09, partial η^2^ = 0.04.

**TABLE 3:** ANCOVA of the SDMT and c-SDMT with the fixed factor age, the covariate sex, and the dependent variable c-SDMT average time/trial and the SDMT final score, respectively.

Source	*df*	*F*	*p*	partial η^2^
**SDMT**				
Corrected model	2	39.45	<0.001	0.49

Sex	1	2.65	0.11	0.03

Age group	1	77.33	<0.001	0.48
Error	83	–	–	–

**c-SDMT**				
Corrected model	2	30.82	<0.001	0.43

Sex	1	2.99	0.09	0.04

Age group	1	59.70	<0.001	0.42
Error	83	–	–	–

*SDMT, Symbol Digit Modalities Test; c-SDMT, computerized Symbol Digit Modalities Test.*

### Within-Test Dynamics: Changes of Time Needed per Trial Across the Sequence of Eight Trials in the c-SDMT

In the repeated measures ANOVA (Table 4) the Greenhouse-Geisser estimate of sphericity showed a substantial deviation (ε = 0.65); therefore, multivariate test results are reported. The time needed to complete a trial varied significantly across the sequence of all eight trials, *V* = 0.59, *F*(7,78) = 16.16, *p* < 0.001, partial η^2^ = 0.59. However, the time needed to complete a trial in the sequence of eight trials did not differ significantly between the younger and older age group, *V* = 0.18, *F*(7,78) = 1.78, *p* = 0.10, partial η^2^ = 0.14.

**TABLE 4:** Repeated measures ANOVA for c-SDMT trials one to eight with age as a between-subject factor.

Effect		*V*	*F*	Hypothesis *df*	Error *df*	*p*	partial η^2^
Trials	Pillai’s Trace	0.59	16.16	7.00	78.00	<0.001	0.59
Trials * Age Group	Pillai’s Trace	0.14	1.78	7.00	78.00	0.14	0.14

### Normative Values of the c-SDMT and Comparison of the Swiss and Canadian Cohorts

**FIGURE 2:**
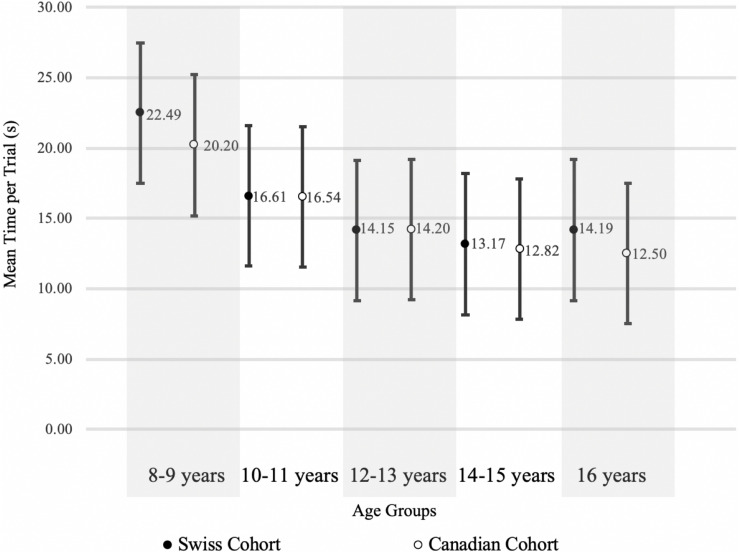
Comparison of c-SDMT mean time per trial and standard deviation of the Swiss and Canadian Cohort.

Normative values for the Swiss and Canadian cohorts are shown in Table 5. The *t*-tests comparing the difference in the mean time per trial revealed no significant difference between the Swiss and Canadian Cohort for any age group. The mean time per trial for each age group of the Swiss and Canadian cohort and the corresponding standard deviation is displayed in Figure 2, which supports the findings of the *t*-tests.

**TABLE 5:** Age normative values in seconds for the Swiss and the Canadian sample and the statistical difference of the mean time per trial between the two samples.

Swiss Sample					
Age group	8–9 years (*n* = 17)	10–11 years (*n* = 23)	12–13 years (*n* = 21)	14–15 years (*n* = 18)	16 years (*n* = 7)
Mean time per trial	22.49 (3.61)	16.61 (2.37)	14.15 (2.26)	13.17 (1.48)	14.19 (3.51)
Trial 1	26.80 (7.77)	18.32 (4.04)	16.53 (4.01)	14.67 (1.89)	17.16 (4.77)
Trial 2	24.04 (4.85)	17.31 (3.74)	15.27 (3.81)	13.83 (1.82)	15.77 (4.10)
Trial 3	23.62 (5.48)	18.31 (3.18)	14.45 (2.46)	14.77 (2.94)	13.64 (3.38)
Trial 4	21.82 (5.66)	17.05 (3.28)	13.88 (2.01)	13.38 (2.38)	13.81 (5.01)
Trial 5	21.32 (4.37)	16.03 (2.60)	14.24 (3.02)	12.92 (1.83)	14.04 (3.39)
Trial 6	22.22 (5.06)	15.31 (2.81)	12.94 (2.38)	11.81 (1.85)	14.09 (3.92)
Trial 7	17.93 (2.63)	14.90 (3.39)	12.82 (2.78)	11.91 (2.56)	12.24 (2.71)
Trial 8	22.08 (7.06)	15.62 (3.17)	13.06 (2.37)	12.20 (1.49)	12.74 (3.27)

**Canadian Sample**					

**Age group**	**8–9 years (*n* = 89)**	**10–11 years (*n* = 116)**	**12–13 years (*n* = 105)**	**14–15 years (*n* = 93)**	**16 years (*n* = 47)**

Mean time per trial	20.20 (3.95)	16.54 (3.46)	14.20 (2.61)	12.82 (1.55)	12.50 (2.28)
Trial 1	21.12 (5.53)	17.03 (3.84)	15.32 (3.50)	13.92 (1.89)	13.57 (2.82)
Trial 2	20.45 (4.78)	17.05 (4.71)	14.61 (3.39)	13.04 (2.03)	12.57 (2.40)
Trial 3	19.99 (4.67)	16.47 (4.01)	13.97 (3.21)	12.81 (2.13)	12.10 (2.61)
Trial 4	19.61 (4.70)	15.77 (3.64)	13.90 (3.48)	12.64 (1.91)	12.21 (2.11)
Trial 5	19.84 (4.68)	16.42 (4.83)	13.85 (2.91)	12.46 (2.07)	12.40 (2.56)
Trial 6	19.55 (4.80)	16.58 (4.17)	13.78 (2.88)	12.44 (2.30)	11.98 (4.49)
Trial 7	19.75 (5.73)	16.20 (3.85)	13.81 (3.31)	12.42 (2.16)	12.75 (4.36)
Trial 8	20.77 (6.03)	16.88 (4.72)	14.32 (3.59)	12.84 (2.09)	12.50 (2.28)
Statistical difference (*p*)	0.53	0.12	0.67	0.76	0.16

*Standard deviations are given in parentheses; n = subsample size. Canadian data was provided by the study team of Bigi et al. (2017).*

## Discussion

The main finding of this study is that both the SDMT and c-SDMT have excellent reliability. However, both tests showed small, but clinically significant practice effects. Furthermore, age had a significant effect in both tests. In the c-SDMT, however, younger and older participants did not differ in the increase of speed within the test, i.e., from trial 1 to trial 8. Sex showed no statistically significant effects.

Our data shows high reliability, both in the SDMT and the c-SDMT, with our ICC values of 0.89 and 0.90, respectively, being comparable to those found in earlier studies [SDMT ICC = 0.72–0.98 (Koh et al., 2011; Pereira et al., 2015; Tang et al., 2018), c-SDMT ICC = 0.92–0.97 (Akbar et al., 2011; Bigi et al., 2017)]. Furthermore, the test was equally reliable in younger and older participants. However, the 95% CIs for the ICCs in our study were quite wide. This can be explained by the small size of our cohort.

As expected, both tests showed small practice effects from test to retest validating the findings of Koh et al. (2011) for the SDMT. However, we found a slightly larger effect in the c-SDMT than in the SDMT. To assess the clinical significance of such effects, the RCIp was calculated. For the SDMT our results (RCIp = −3.18, 14.01) are comparable with values reported by Koh et al. (2011) (RCIp = −5.29, 10.89) and Tang et al. (2018) (RCIp = −7.2, 12.4). For the c-SDMT no such values have been published in the literature. Our calculations suggest a RCIp of −5.45, 1.46. However, for broader clinical use, further validation in larger cohorts is needed to confirm this value.

As expected, age proved to have a significant effect on the results of both the SDMT and the c-SDMT. When looking at the norm values calculated for age groups with 2-year age bands (Table 5) in the c-SDMT, older participants performed faster than younger ones, confirming that processing speed increases with age. This is also shown in Figure 2, where the test performance of our cohort is comparable to that of the Canadian cohort (Bigi et al., 2017). The repeated measures ANOVA showed that there is a significant effect from trial one to trial eight in the c-SDMT. These findings are consistent with the results of earlier studies (Akbar et al., 2011; Bigi et al., 2017). The improvement of performance in successive trials did not differ significantly between the younger and older age group. We interpret this as a result of the simple test design of the c-SDMT, which does not necessitate the use of complex strategies. If such strategies were necessary, we would have expected the older group to start at a higher level, adapting faster to the testing procedure. Therefore, they would improve less in the successive trials than the younger group, which would need more trials to develop such strategies.

The ANCOVA showed no significant effect of sex (Table 3). Roivainen (2011) suggests that a minimum sample size of *n* = 100 is needed when evaluating sex effects. Therefore our results, especially those of the c-SDMT with a *p*-value of 0.09, must be interpreted with caution.

When comparing data on the c-SDMT performance of the Swiss cohort to that of the Canadian cohort (Bigi et al., 2017), we found no significant difference in the mean time per trial in any age groups (Table 5). Although the Swiss cohort was much smaller (*n* = 86) than the Canadian cohort (*n* = 478), the standard deviations show a similar range (Table 5 and Figure 2). These findings suggest that the influence of cultural and linguistic differences between the Swiss and Canadian cohorts are negligible, further validating our data.

There are several limitations to this study. Firstly, we had a relatively small cohort of neurologically healthy participants. While we could establish a very good concurrent validity with the written SDMT, the study design did not allow the estimation of predictive validity of the c-SDMT in terms of every day functioning of the participants. Currently, we are conducting a study to establish predictive validity of the c-SDMT in a range of patient samples. Furthermore, the administration of the two implemented tests was not controlled. Finally, all conclusions regarding test-retest reliability (ICC, RCI) are limited to the test-retest interval of 14 days as applied in the study and my change with longer or shorter retest intervals. However, our study had the aim of validating the c-SDMT in a Swiss population knowing that there already exists a large database of Canadian children and adolescents. For further validation, studies that include participants with neurological conditions are needed.

In conclusion, the c-SDMT is a valid alternative to standard paper-and-pencil tests of processing speed. It is a promising bedside tool to track the short-term development of neurological conditions associated with motor impairment, such as traumatic brain injury and epilepsy. Further validation studies should include this specific patient population to confirm this hypothesis.

## Data Availability Statement

The raw data supporting the conclusions of this article will be made available by the authors, without undue reservation.

## Ethics Statement

The studies involving human participants were reviewed and approved by the Gesundheits- und Fürsorgedirektion des Kantons Bern; Kantonale Ethikkommission für die Forschung; Murtenstrasse 31; 3010 Bern; Switzerland. Written informed consent to participate in this study was provided by the participants’ legal guardian/next of kin.

## Author Contributions

SR and CH performed the acquisition of data, analysis and interpretation of data, and draft of the manuscript. UJ-R, M-NK, MS, and LC were involved in the data acquisition. SB and KL drafted and supervised the study and together with BB and AF critically revised the manuscript for intellectual content. All authors were involved in the conceptualization, design of the study, read, and approved the manuscript.

## Conflict of Interest

The authors declare that the research was conducted in the absence of any commercial or financial relationships that could be construed as a potential conflict of interest.

## Abbreviations

c-SDMT: computerized Symbol Digit Modalities Test
IPS: information processing speed
SDMT: Symbol Digit Modalities Test.
